# A CD1c lipid agnostic T cell receptor bispecific engager redirects T cells against CD1c^+^ cells

**DOI:** 10.3389/fimmu.2025.1614610

**Published:** 2025-07-24

**Authors:** Rita Szoke-Kovacs, Sophie Khakoo, Victor Lopes Rangel, Pietro Della Cristina, Johanne Pentier, Rahul Khanolkar, Sam El-Ajouz, Robert Simmons, David K. Cole, Peter Gogolak, Mariolina Salio, Vijaykumar Karuppiah

**Affiliations:** ^1^ Experimental Immunology, Immunocore Ltd, Abingdon, United Kingdom; ^2^ Department of Immunology, University of Debrecen, Debrecen, Hungary

**Keywords:** T cell receptor, bispecifics, T cell engager, CD1c, lipids, leukemia, immunotherapy

## Abstract

**Introduction:**

Immunotherapy is emerging as an efficacious treatment for some cancers, complementing traditional chemo-radiation therapies. Specific markers at the cell surface of cancer cells can be used as immunotherapy targets. However, many of these markers are defined by a patient’s genetic background, limiting their use across the human population.

**Methods:**

Here, we investigated the non-polymorphic antigen presenting molecule, CD1c, that is only expressed on subsets of mature hematopoietic cells, as a potential immunotherapy target with reduced risk of off-tumor on-target toxicity in healthy tissues.

**Results and discussion:**

We identified a T cell receptor (TCR) which recognises CD1c in a lipid independent manner and determined the crystal structure of the TCR-CD1c complex which revealed flexibility around the lipid binding region, and a new binding mechanism of auto-antigen recognition. We generated affinity enhanced variants of the TCR and fused them to an anti-CD3 antibody for T cell redirection. Lipidomic analysis revealed promiscuous lipid recognition of CD1c by the affinity enhanced TCR variants, with preference for larger lipid head group, a finding which is supported by the crystal structure. The bispecific molecule induced potent re-directed T cell killing of CD1c positive cell lines. These proof-of-concept findings demonstrate that CD1c targeting TCR bispecific engagers might be good candidates for the development of non-MHC restricted, universal therapeutics for the treatment of CD1c+ leukemias.

## Introduction

T cell receptor (TCR) recognition of peptide-human leukocyte antigens (pHLA) molecules can initiate potent T cell responses against infected or neoplastic cells. This has led to the development of several cellular- and protein-based therapeutics that target tumor associated pHLAs (1). However, the genes that encode HLA molecules are extremely polymorphic, which limits treatment eligibility because of the patient’s genetic background (2). Indeed, the majority of HLA targeting therapeutics are focused on the most frequent Caucasian HLA allele, HLA-A*02:01 (expressed by ~47% of Caucasians) (3).

The cell surface glycoproteins CD1 (cluster of differentiation 1), are a family of non-polymorphic antigen presenting molecules that present lipid antigens, and include the group 1 (CD1a, b and c), and group 2 (CD1d) CD1 molecules (4). Most lipid antigens presented by CD1 molecules consist of hydrophobic acyl chains and hydrophilic head groups. Generally, the hydrophobic acyl chains are anchored in the CD1 binding pocket created by the α1-α2 domains whereas the hydrophilic head groups are protruding at the CD1 surface, where they are available for recognition by the TCR (5). CD1 isoforms differ in their cytoplasmic tails, which influences their intracellular trafficking, and in the size and shape of the antigen binding groove (6), which determines the repertoire of lipids bound to each isoform. The structure of CD1c bound to mycobacterial mycoketides revealed distinctive features of the CD1c antigen binding groove (7, 8) Specifically, the CD1c A’ pocket is continuous with the F’ pocket, creating a structure that opens externally through accessory gaps called the D’ and E’ portal (6). This feature permits more diverse hydrocarbon chain lengths to be accommodated in the A’ pocket. In the same crystal structure two lipids were mapped to the CD1c binding groove, the methylated mycoketide backbone in the A’ pocket and a C12 hydrocarbon chain in the F’ pocket, while the phosphate or phosphomannose head groups extended out of the F’ portal (6). This unique architecture allows the presentation of a wide array of lipid-antigens that can be recognised by autoreactive and microbial reactive T cells.

The non-polymorphic nature of the CD1-family of molecules (i.e. they are identical in all humans) means that T cell responses towards these molecules are not restricted to the donor’s genetic background. Moreover, as lipids have a low mutation rate, there is a lower risk of tumour escape when targeting lipid antigens compared with peptide-HLA. However, it remains to be understood to what extent CD1-restricted TCRs are specific for an individual lipid, or a lipid family. Indeed, there is evidence for both lipid-dependent and lipid-independent recognition of CD1 molecules (9), raising important questions concerning the nature of self-reactivity and pathogen targeting of CD1-restricted T cells.

Unlike HLA class I molecules, that are expressed on virtually all nucleated cells, CD1c is only expressed on cells of hematopoietic origin, such as thymocytes and mature hematopoietic cells (e.g. B-lymphocytes and myeloid/monocytic antigen presenting cells, APCs) (10). Furthermore, CD1c is not expressed on hematological precursor cells, so any treatment that targets CD1c expressing cells would allow for repopulation of these cells from the bone marrow. This makes CD1c an ideal target for hematological cancers, as its restricted expression would limit toxicity in other healthy tissues. Indeed, it has been demonstrated that CD1c-restricted T cells can target leukemia cell lines in a CD1c-dependent manner while sparing CD34^+^ stem cells, and CD1c has been shown to be expressed in AML, T-ALL, B-ALL blasts and non-Hodgkin lymphomas in 50-75% of patients (11).

To understand the breadth and ligand specificity of CD1c reactive T cells, we identified CD1c restricted T Cell Receptors (TCRs) by phage panning. We then solved the co-complex crystal structure of a CD1c-restricted TCR that could bind to a broad CD1c-bound lipid-repertoire, revealing the molecular basis of lipid-agnostic TCR recognition of CD1c. Using a structurally guided approach, we generated affinity enhanced variants of the TCR and fused them to an anti-CD3 antibody to create CD1c-targeting bispecific T cell engagers. The bispecific T cell engagers were able to mediate potent re-directed T cell killing of CD1c positive target cell lines. We validated through mass spectrometry lipid agnostic recognition of CD1c by one of the bispecific engagers. These proof-of-concept findings demonstrate that CD1c-targetting T cell engagers could be developed as novel therapeutics for hematological cancers, bypassing the limitations of HLA restriction.

## Materials and methods

### Panning using TCR libraries

TCR libraries were generated as previously described by Coles C et al. (12). Biotinylated mammalian expressed CD1c molecules expressing the endogenous repertoire of self-lipids (thereafter referred to as CD1c-endo) were captured on streptavidin-coated paramagnetic beads and incubated with the library of purified phage particles pre-blocked in 3% MPBS buffer. Phage particles were eluted in trypsin and used to infect early log phase TGI *E. coli* cells and plated onto YTEag plates at 30°C for 16 h. Three rounds of selection were performed.

### TCR engineering

To obtain CD1c-restricted, affinity-enhanced TCRs, mutations were introduced to the TCR (S2c^WT^) and the libraries were panned using a phage display method adopted form Li et al. (13). Phage panning was performed in detergent free buffer and the final re-suspension and blocking steps were carried out in 2% BSA containing PBS to avoid the loss of lipid ligands. A panel of high-affinity TCR mutants were obtained with mutations in the α and/or the β chain (data not shown). One TCR mutant (S2c^a5b6^) derived from this panel was selected for further study.

### Construct design, protein expression and purification

The TCR variants were cloned into the pGMT7 vector and expressed in the BL21 (DE3) Rosetta pLysS *E. coli* strain as described previously (14, 15). TCR constructs for biophysical analysis were designed to include the variable and constant domains of both chains (α and β) with an engineered inter-chain disulphide bond as previously described (16). To generate bispecific T cell engagers (thereafter called ImmTAC molecules, total size ~75kDa) (17), the TCRβ chain was fused at its N-terminal to the UCHT1 anti-CD3 scFv fragment through a single GS (GGGGS) linker. The sequences of the anti-CD3 scFv and the linker used are described in detail in patent 13/319597. CD1c and TCR fused to anti-CD3 scFv expression cassettes were cloned into pCDNA3.1, and proteins were expressed in mammalian cells using the Expi293F Expression System (Thermo Fisher Scientific).

### Molecular cloning and expression of CD1c molecules

The human CD1c protein α1, α2 and α3 domains were linked at the N-terminal to the human β2-microgobulin (β2m) via a glycine-serine linker (GGGGSGGSGSGGGSS) followed by C-terminal Avi-TagTM and 6xHis tag and were synthesized as a single chain construct (GeneArt, ThermoFisher Scientific) and subcloned into pCDNA3.1 episomal expression vector (Invitrogen). The vector was engineered to express the BirA enzyme for biotinylation of the recombinant CD1c protein. Expi293F cells (ThermoFisher Scientific A14527) were transfected with 1µg/ml plasmid following the ExpiFectamine™ 293 Transfection Kit protocol (Life Technologies A14636). Mannosidase I enzyme inhibitor Kifunensine (Merck K1140-1MG) was used to obtain homogeneous high mannose type N-linked glycans. Biotinylated CD1c monomers loaded with endogenous lipids (CD1c-endo) were expressed and secreted into the cell medium for five days post-transfection and purified by Ni-Affinity chromatography on the HisTrap excel (5ml) column (Cytiva 17371205), followed by Size Exclusion chromatography on the Superdex200 increase column (Cytiva 28990944). The CD1c proteins were PNGAseF (NEB P0704S) treated to remove glycans.

### ImmTAC redirection assays

The activity of the ImmTAC molecules was tested through their ability to redirect T cells against a range of antigen-positive and antigen-negative cell lines, as previously described (18). Targets were THP1 cells, THP1 overexpressing CD1b or CD1c, and THP1 cells lacking β2m and CIITA (THP1 DKO), hence devoid of MHC class I, class II and CD1 molecules (19); C1R, C1R overexpressing CD1c or CD1d, K562 and K562 overexpressing CD1c, SKW3, OCIM1, HPB-ALL, NALM6, CCRFSB, MOLT4 (**Supplementary Table S1**). Cells were grown in RPMI 10% FCS, supplemented with glutamine, sodium pyruvate, Hepes, non-essential amino acids and pen streps (all from Gibco).

Blood was purchased from NHSBT and PBMCs were isolated from blood by density-gradient centrifugation. CD14^+^ monocytes, CD19^+^ B cells, and CD2^+^ T cells were purified using Miltenyi MicroBeads. The target and effector cells were co-cultured in 1:1 E:T ratio, in the presence of a dilution series (0.001pM) of ImmTAC molecules in RPMI 10% FCS supplemented with glutamine and pen strep. The assay plates were incubated at 37°C, 5% CO2 for O/N (21h). Specificity of the ImmTAC was tested in the presence of 10μg/ml blocking CD1c antibody (clone L161, Biolegend) or a relevant isotype control. The cells were stained for CD1c expression (Biolegend clone L161), CD1d expression (Biolegend clone 51.1) or CD1b expression (Biolegend clone SN13). T cell activation markers (Biolegend CD25 clone M-A251 and CD137 clone 4B4-1) were assessed on CD4 and CD8 T cells by flow cytometry on a BD Fortessa X20 instrument equipped with five lasers. Data were analysed with Flowjo.

Experiments with human PBMCs were performed in accordance with Immunocore HTA licence.

### MAGPIX peptide screening

To evaluate the cross-reactivity of TCRs, a method utilizing a MagPLEX bead kit (Invitrogen, UK) was employed as described before (20). The TCRs were exposed to a diverse range of HLA-peptide complexes and CD1-lipid complexes, allowing for a thorough examination of the specificity and selectivity of the TCRs. Biotin-tagged HLA-A*02:01, or HLA-A*01:01 were refolded with common peptides, and CD1 molecules loaded with endogenous lipids were expressed in Expi293F cells. Phagemid-encoded TCR molecules were displayed on the surface of bacterial virus M13 and incubated with biotinylated self-peptide-HLA complexes or all the CD1 isoforms immobilized on neutravidin-conjugated MagPLEX™ magnetic beads. Phage-specific PE-conjugated anti-M13 bacteriophage coat protein g8p antibody [RL-ph2, 2B Scientific Limited MUB0604 and conjugated in house to R-Phycoerythrin using a Conjugation kit (Abcam ab102918)] allowed the identification of positively bound beads by MAGPIX analysis. Positive binders elicited a fluorescent signal above the background. The background level was established by calculating three times the median intensity of the bead regions bound to the native helper-phage. Positive binding was calculated as a percentage of the signal from binding to the index peptide. The mean value of triplicate measurements for each interaction was calculated in every experiment.

### SPR single cycle kinetic analysis

The binding analysis of the purified TCRs was performed either using a BIAcore^®^ T-200 with CM5 sensor chip (Cytiva 29149604) (for Steady State affinity), or BIAcore^®^ 8K (GE Healthcare) with Series S CM5 sensor chip (Cytiva 29149603) (for Single Cycle kinetics). Streptavidin molecules [~5000 response units (RU)] were then linked covalently to the chip surface by amine coupling. Approximately 100-1000 RU of CD1c-endo molecules were attached to the CM5 sensor chip at a slow flow rate of 10 μL/min. One flow cell was left blank and used as a negative control. The TCRs and ImmTAC molecules were concentrated to 100µM, and 8 serial dilutions (1/2) were injected over the sensor chips at 25°C at a flow rate of 30 μL/min. Results were analyzed using Biacore Insight Evaluation (GE Healthcare) and GraphPad Prism. The equilibrium binding constant (KD) values were calculated assuming a 1:1 interaction by plotting specific equilibrium-binding responses against protein concentrations followed by non-linear least squares fitting.

### CD1c-TCR co-purification for lipid mass spectrometry

CD1c-endo was with S2c^WT^ or S2c^a5b6^ ImmTAC molecules at 1:1 ratio for lipidomic analysis. The complexes were purified by Size Exclusion chromatography on Superdex200 increase column (Cytiva 28990944) to separate TCR-bound CD1c-lipid complexes from non-bound.

### X-ray crystallography

S2c^WT^- and S2c^a5b6^ were mixed with CD1c-endo at 1:1.1 (TCR: CD1c) molar ratio, concentrated to ~10 mg/ml and buffer exchanged into 10 mM Tris pH 8.0 and 20 mM NaCl. Crystallisation plates containing sitting drops of 150 nl of protein complex and 150 nl of reservoir solution were set up using Gryphon crystallisation robot (Art Robbins Instruments) and incubated at 20°C. Crystals appeared in the following conditions for the complexes:

S2c^WT^-CD1c: 0.1 M Sodium cacodylate trihydrate pH 6.4, 0.2 M Calcium chloride dihydrate, 16% (w/v) PEG 3350 and 3% (w/v) 1,6-Hexanediol.

S2c^a5b6^-CD1c: 0.2 M Sodium thiocyanate and 20% (w/v) PEG 3350.

The X-ray diffraction data were collected at the Diamond Light Source (UK) beamlines I03 and I04. Data were integrated and scaled using the autoPROC staraniso (21) and xia2 3dii (22) processing pipelines for S2c^WT^-CD1c and S2c^a5b6^-CD1c respectively. The structure of S2c^WT^-pHLA complex was solved by molecular replacement using the PDB codes 3OV6 (for CD1c-B2m), 4P4K (for TCRα) and 5FK9 (for TCRβ) as search models in Phaser (23) within the CCP4 suite (24, 25). The S2c^a5b6^-CD1c complex was solved by molecular replacement using a single copy of the S2-awtbwt-CD1c complex as the search model. The models were built using iterative cycles of manual model building in COOT (26) and refinement using Refmac (27). The stereochemical properties and validation of the models were assessed using Molprobity (28). Data collection and refinement statistics are given in **Supplementary Table S2**. The structural figures were generated using Pymol (Schrödinger).

### Mass spectrometry analysis of the CD1c-associated lipids recognised by CD1c-ImmTACs

Mass spectrometry-based lipid analysis was performed as previously described by Szoke et al. (29). Briefly, lipid mass spectrometry of the purified protein-lipid complexes was carried out at Lipotype GmbH (Germany), as described by Surma et al (30). After extraction, the lipids were analysed by mass spectrometry using lipid class-specific internal standards. The mass spectra were acquired in a single acquisition in positive and negative ion mode, with a resolution of Rm/z=200 = 280000 for MS and Rm/z=200 = 17500 for MS-MS experiments. Lipids were identified based on the molecular masses of the intact molecules in the MS mode and using both the intact masses and fragment masses in the MS-MS mode. The lipid identifications were filtered based on mass accuracy, occupation threshold, noise and background followed by normalization and statistical analysis. Lipids were quantified by normalizing the peak intensities to the intensity of the lipid class-specific internal standards. The data was filtered to lipids with a signal-to-noise ratio of <5, and a signal intensity 5-fold higher than in corresponding blank samples. The pmole values of the individual lipid molecules (species of subspecies) were summed to yield the total amount of the lipid class. The pmole values of the lipid classes were then normalized to the total lipid amount yielding mol% per total lipids. For lipid classes that are analysed semi-quantitatively, the peak intensities were normalized to the intensity of an internal standard which does not belong to the respective lipid class (normalized intensities). The normalized intensities were further standardized to total lipid content of each sample (normalized relative abundance). Bioinformatics analysis was done in-house using R workflow (R version R-4.3.2).

### Data filtering and HLA-A2 background

In total, mass spectrometry identified 813 lipid features across the samples, 101 of which were also present in the HLA-A2 controls. These accounted for 184 pmoles of total lipid, with 104 pmoles comprising four unique lipid features (GM1 40:2;2, GM1 42:1;2, GM1 42:2;2, GM3 42:2;2). Lipids detected in ImmTAC-only samples contributed only 2-4% of those in the ImmTAC-CD1c complexes. The most abundant lipid feature in the ImmTAC-only samples (GD2 42:1;2) was not detected in the ImmTAC-CD1c complexes but was present in the unbound CD1c fraction. GM3 42:2;2 was similarly detected in ImmTAC-only, unbound CD1c, and ImmTAC-CD1c complexes, identifying it as a false positive. After accounting for these features, the overall background was reduced to negligible levels (~0.4%).

## Results

### Structural basis of CD1c-endo recognition by the S2c^WT TCR^

CD1c restricted TCRs described to date bear the αβ or the γδ chains and can be either self-reactive [like the 3C8 TCR (31)] or microbial specific (for example to mannosyl phosphomycoketides (8). To further characterise the breadth and specificity of the CD1c-restricted T cell repertoire, we isolated a CD1c-restricted TCR (S2c^WT^) from in-house TCR phage libraries. The S2c^WT^ bound to CD1c molecules loaded with endogenous lipids (CD1c-endo) and purified from human embryonic kidney (HEK) cells with an average affinity (K_D_) of 16.7 μM (as measured by surface plasmon resonance) (**Supplementary Figure S1A**), which is in the typical range for classical TCR-pHLA interactions (14, 32, 33). To gain insight into whether the S2c^WT^ TCR was lipid specific, we estimated the theoretical Rmax based on the amount of CD1c-endo molecules loaded onto the chip surface. On average, the S2c^WT^ TCR reached around 75% of the theoretical Rmax, suggesting a relatively lipid-independent binding mode, differing from previously published data for other CD1c-reactive TCRs (31) (**Supplementary Figure S1A**).

**Figure 1:**
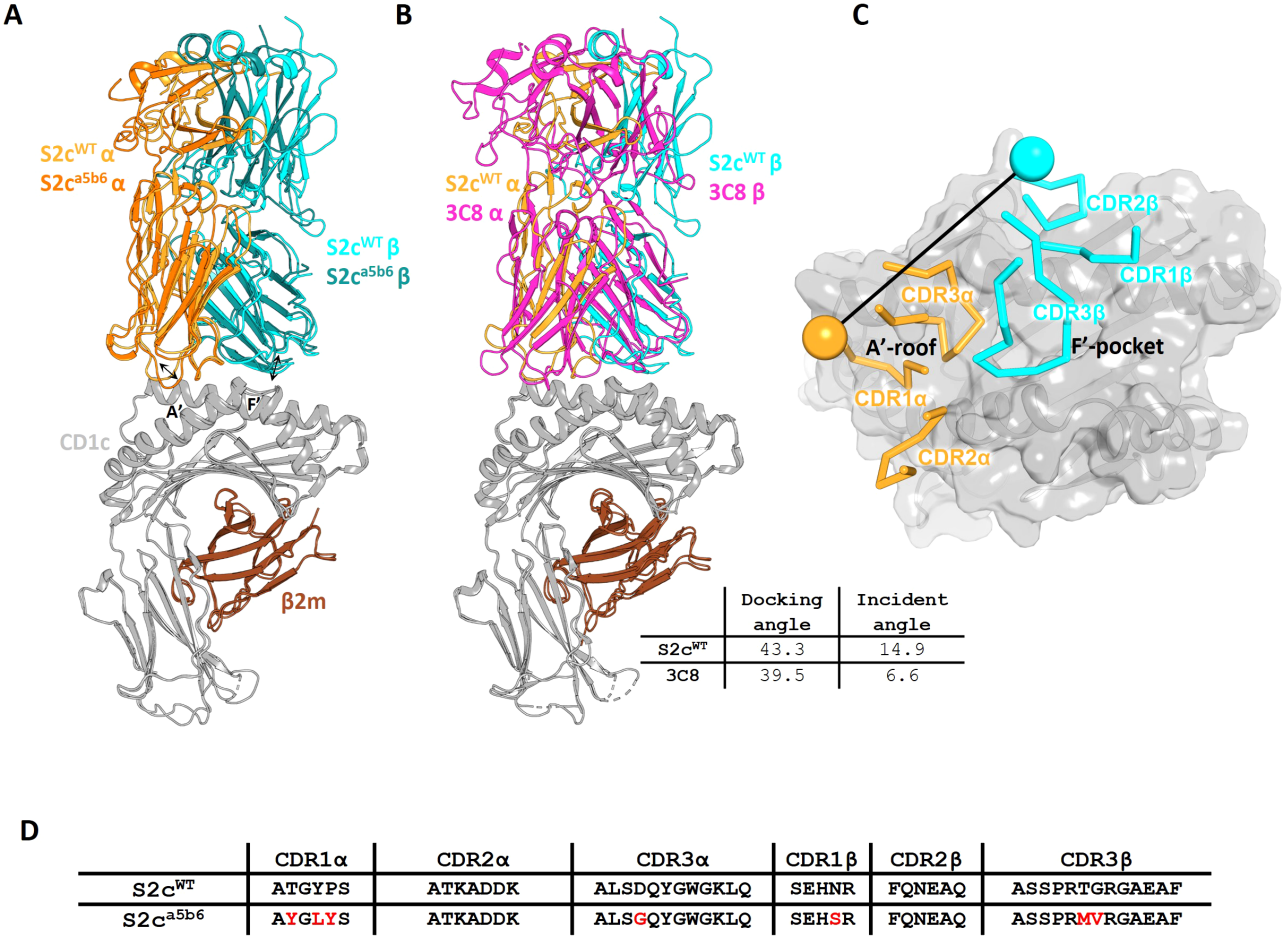
Crystal structures of CD1c-S2c TCR complexes. **(A)**. Cartoon overlay of phage-isolated S2c^WT^ and high affinity S2c^a5b6^ TCRs in complex with CD1c-endo. TCRα, TCRβ, CD1c and β2m chains are coloured as orange, cyan, grey and brown respectively. The S2c^a5b6^ TCR chains are coloured in darker shades. The arrows indicate the changes in CDR1α and CDR3β loop conformations between S2c^WT^ and S2c^a5b6^. **(B)**. Cartoon overlay of CD1c-S2c^WT^ and CD1c-3C8 TCR (PDB) complexes. The 3C8 TCR chains are coloured in magenta. **(C)**. Top view showing the CD1c as grey surface and S2c^WT^ CDRs as ribbons. The position of disulphide bonds in the TCR variable domains are indicated by spheres and the line connecting them represents the vector used for calculating the docking angle. **(D)**. CDR sequences of S2c^WT^ and S2c^a5b6^ TCRs. Mutations introduced in S2c^a5b6^ variant are highlighted in red. TCR chains: S2c (TRAV9-2*02/TRBV7-9*01); 3C8 (TRAV29/TRBV 7-2).

To better understand the molecular basis for these binding characteristics, we solved the structure of the S2c^WT^-CD1c-endo trimolecular complex to 2.04Å (**Supplementary Table S2** **).** The TCR bound to CD1c centrally (**Figure 1A**), with a similar diagonal docking angle (43.3°) compared to the previously reported CD1c-restricted 3C8 TCR (31) (**Figure 1B**), with the CDR1 and 2 loops positioned over the CD1c α-helices and the CDR3β loops directly over the CD1c F’-portal (**Figures 1C, D**), seemingly primed to make contacts with the lipid head group.

This binding mode is most closely related to the known structures of CD1b-restricted TCR complexes (34), while the CD1a-restricted TCRs structures resolved to date have unique binding modes (35), and CD1d-restricted TCRs have been shown to either be predominantly A’-roof focused, or F’-portal focused (36, 37) (**Supplementary Figure S2**).

**Figure 2:**
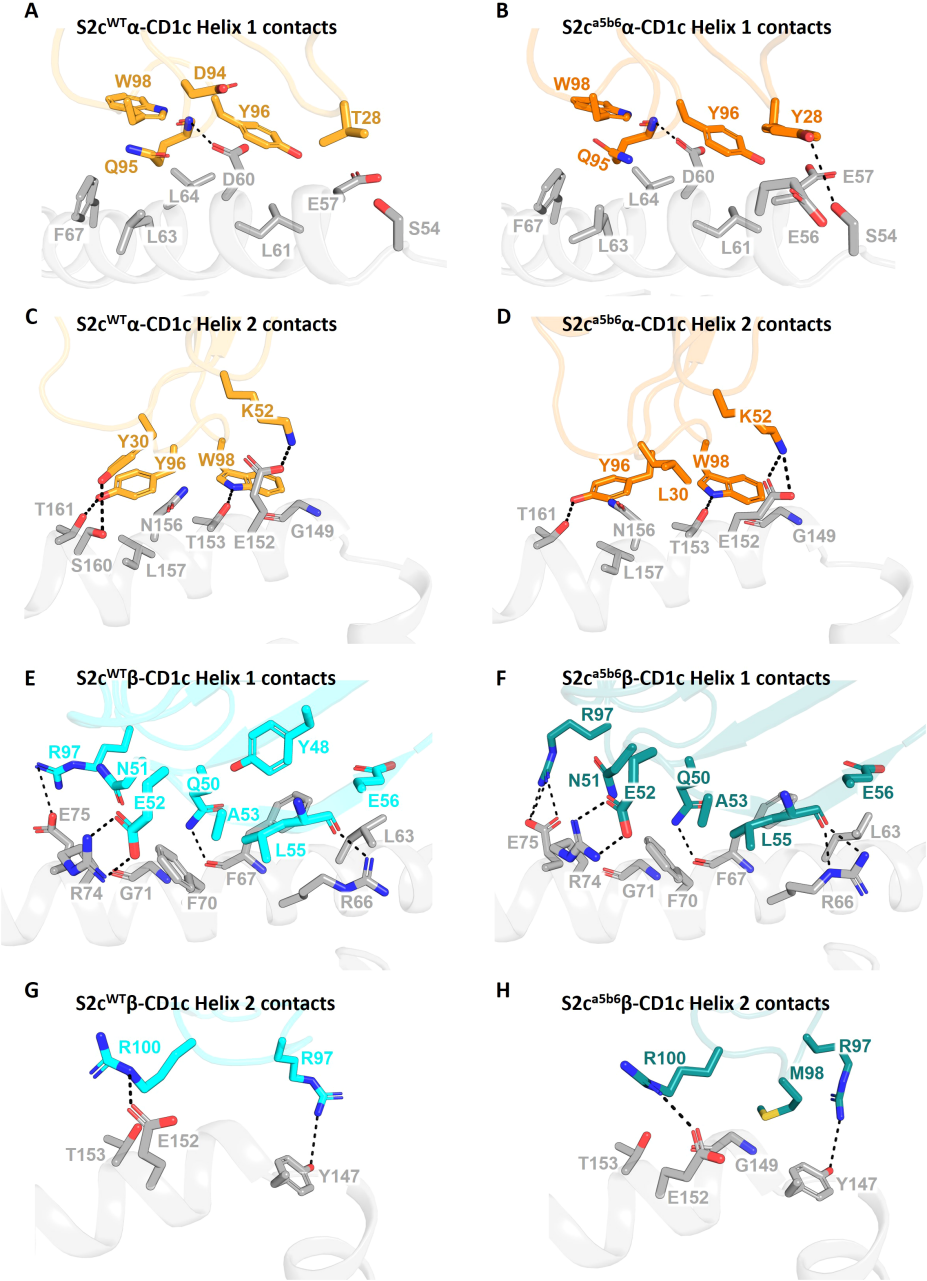
Comparative interaction analysis of S2c^WT^ and S2c^a5b6^ TCRs with CD1c-endo. Side-by-side comparison of residues from the S2c^WT^ and S2c^a5b6^ TCRs interacting with the helices of CD1c-endo. **(A, C)** The S2c^WT^ TCR α-chain (orange), dominated by CDR3α, forms substantial contacts along the length of CD1c helices 1 and 2 (grey), respectively. **(B, D)** The S2^ca5b6^ TCR α-chain (darker orange) engaging CD1c helices 1 and 2, respectively. Y28α and L30α form additional van der Waals contact with CD1c. **(E, G)** The S2c^WT^ TCR β-chain (cyan) interacting with CD1c helices 1 and 2, respectively, contributed by CDR2β and CDR3β residues. **(F, H)** The S2c^a5b6^ TCR β-chain (darker cyan) interacting with CD1c helices 1 and 2, respectively. M98β makes additional van der Waal interactions with CD1c. Interface residues within 4 Å are shown as sticks. Dotted lines denote polar contacts.

Analysis of the S2c^WT^ TCR footprint demonstrated that most contacts were focused over the CD1c A’-roof, with substantial contacts along the length of the CD1c helix 1. This binding footprint resulted in a buried surface area of 1015 Å, within the normal range for TCR-pHLA and other TCR-CD1 interactions (11, 38), with the TCR α-chain contributing to just over half of the interface. Interface analysis demonstrated that the TCR α-chain dominated contacts, mediated largely by interactions between the CDR1α and CDR3α loops with both the CD1c helices. TCR β-chain contacts were mostly driven by CDR2β to CD1c helix 1, with additional contacts from CDR3β to both helices and CDR1β making no contribution to the interface (**Figure 2**).

**Figure 3:**
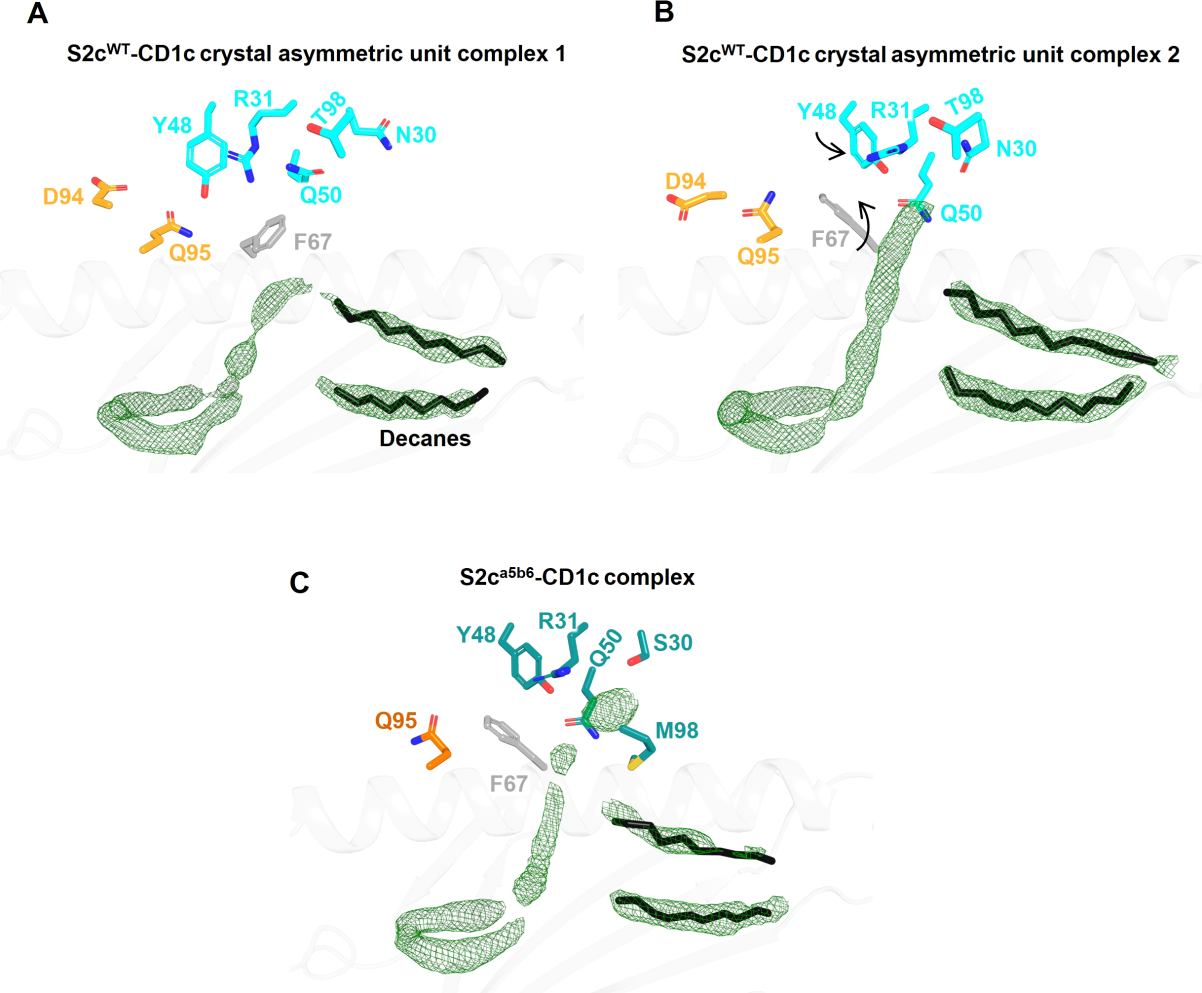
Comparison of Fo-Fc electron density in the lipid binding pockets of CD1c-S2c TCR structures. **(A, B)** Lipid binding region of the S2c^WT^-CD1c complex showing variations in lipid density within the two asymmetric unit molecules in the crystal structure. CD1c F67 side chain conformation flip and associated TCR Y48β displacement are indicated by arrows **(B)**. TCR residues that are near the F-pocket and conformational differ between the two asymmetric unit molecules are displayed as sticks. The Fo-Fc omit map (green mesh) is contoured at 3σ. The spacer lipids (decanes) are shown as black sticks. **(C)** Lipid binding region of the S2c^a5b6^-CD1c complex with highly similar electron density and conformation of residues within the four asymmetric unit molecules in the crystal structure.

We did not observe clear density for the lipid head group(s), likely because a mixture of lipids is bound to CD1c-endo molecules. Thus, it is possible that the S2c^WT^ TCR made additional contacts with the lipid that were not visible in our structure. In fact, the two copies of the S2c^WT^-CD1c-endo molecule observed in the asymmetric unit of the crystal structure showed significant difference in the density of the lipid head group region (**Figures 3A, B**). This flexibility in accommodating various lipid head groups stems from the two different side chain conformations adopted by CD1c F67, with larger lipid head groups likely requiring F67 to adopt the outward facing conformation. Interestingly, S2c^WT^ TCR was able to bind to both CD1c F67 conformations with a mix of lipid head groups, through concurrent changes in the position of the S2c^WT^ TCR side chain surrounding the CD1c F67 and F’-portal region. In particular, Q95α and Y48β flipped to accommodate the CD1c F67 outward conformation, leading to further changes in R31β, N30β and Q50β to likely interact with the larger lipid head group (**Figure 3B**). Lastly, two additional densities were observed in the CD1c groove which were similar in size to the ‘spacer lipids’ previously noted for other CD1 molecules (31, 39). As previously shown in the literature, modelling and refining of decanes agreed well with the density observed.

Although invariant CD1c restricted TCRs have not been identified, we compared the contact footprint of the S2c^WT^ TCR with the 3C8 TCR, the only other published structure of a TCR-CD1c co-complex (31), to provide insight into any shared binding characteristics. Despite being encoded by different TRAV and TRBV segments, both the S2c^WT^ and 3C8 TCRs interact with similar set of CD1c residues, with the slight difference in docking orientations between them enabling S2c^WT^ to contact more of the CD1c helix 1 residues whereas 3C8 is contacting more of the CD1c helix 2 residues (**Supplementary Tables S3**, **S4**). CD1c residues Asp65, Glu80 and Glu157 play key roles for both TCRs, making multiple van der Waals (vdWs), and electrostatic contacts (hydrogen bonds and/or salt bridges) with S2c^WT^ and 3C8 (5). However, although Gln151, Glu153 and Ser169 are important contact residues for 3C8 (multiple vdWs and electrostatic interactions), they were not contacted by S2c^WT^. Similarly, Arg71 and Leu162 are important contact residues for S2c^WT^, yet were not contacted by 3C8. Thus, consistent with the similar, but not identical, binding modes utilised by the S2c^WT^ and 3C8 TCRs, some conservation of contact interfaces was observed, but was supported by a network of unique contact points for each TCR.

### Affinity enhancement of the S2c^WT^ TCR

Natural TCRs targeting MHC-peptide or lipid CD1 complexes, generally have low affinities, in the 10 to 100 µM range, around 1000-fold lower than typical antibodies. To harness TCRs as soluble therapeutic engagers, their affinity must be enhanced, resulting in molecules that have a longer binding half-life (t_1/2_) (40). The only FDA approved soluble TCR based bispecific against pHLA has pM affinity (41–43), and other known bispecific clinical molecules are in the nM to pM range, significantly higher than natural TCR affinities. To obtain variants with increased affinity for CD1c, the S2c^WT^ TCR was subjected to mutagenesis using phage display libraries as previously described (13).

The phage libraries used for affinity maturation contained mutations to enhance the TCR affinity towards CD1c regardless of the lipid ligand. Indeed, the structure-guided mutations were designed to allow more contact between the TCR α chain and the CD1c heavy chain (helix 1 and 2) and to target direct contacts between the TCR β chain and the CD1c heavy chain (helix 1). We panned libraries with low mutational load in all 3 CDRs of a TCR chain and libraries with high mutational load in a single CDR. Finally, we combined mutations from different libraries to generate high affinity TCR variants.

Eleven TCR variants were identified through two generations of affinity enhancement. We selected a single TCR variant (S2c^a5b6^) with high affinity (K_D_ of 14.5 pM), representing ~1-million-fold affinity enhancement (S2c^WT^ K_D_=16.7 µM), and a t_1/2_ of 9.6 hours, for further investigation (**Table 1**; **Supplementary Figure S1B**). The S2c^a5b6^ TCR did not bind to other CD1-endo complexes or to HLA molecule refolded with common peptide complexes, confirming its specificity and lack of cross-reactivity (**Supplementary Figures S3A, B**).

**Table 1:** Binding dissociation constant of the affinity enhanced TCR variants.

TCR	TRAV	CDR1α	CDR3α	TRBV	CDR1β	CDR3β	T_1/2_ (h)	K_D_ (M)
S2c-a2b1	TRAV9-2	ATGYPS	CALSGQYGWGKLQF	TRBV7-9	SEHNR	CASSPRMVRGAEAFF	0.45	1.05E-09
S2c-a2b6	TRAV9-2	ATGYPS	CALSGQYGWGKLQF	TRBV7-9	SEHSR	CASSPRMVRGAEAFF	3.32	3.05E-11
S2c-a5b1	TRAV9-2	AYGLYS	CALSGQYGWGKLQF	TRBV7-9	SEHNR	CASSPRMVRGAEAFF	4.58	2.97E-11
S2c-a5b6	TRAV9-2	AYGLYS	CALSGQYGWGKLQF	TRBV7-9	SEHSR	CASSPRMVRGAEAFF	9.65	1.45E-11

Binding dissociation constant (affinity) of the affinity-enhanced variants interacting with CD1c loaded with endogenous lipids from HEK cells was measured using surface plasmon resonance.

To understand the molecular details of interactions between the high affinity TCR (S2c^a5b6^) and CD1c-endo, we solved the crystal structure of the S2c^a5b6^-CD1c complex to 2.27 Å resolution (**Figure 1A**). The asymmetric unit contained four molecules of the complex, all of them revealing a lipid density of a very similar size and extending out of the F’-portal, surrounded by the TCRβ residues. In this structure, the CD1c F67 adopts a single outward facing conformation, like the alternate conformation observed for the S2c^WT^-CD1c complex (**Figure 3C**). Also, the side chain conformation of Y48β, R31β and Q50β in S2c^a5b6^ were consistent with the S2c^WT^ alternate conformation. Crucially, S2c^a5b6^ mutated residues S30β and M98β create additional space to accommodate lipid head groups and wrap around the lipid density. These results suggest that the S2c^a5b6^ TCR prefers larger lipid head groups compared to the S2c^WT^ TCR. Importantly, these observations were supported by the mass spectrometry analysis of enriched lipids from the S2c^a5b6^-CD1c complex.

Out of the seven mutations there were introduced, the higher affinity of S2c^a5b6^ towards CD1c was predominantly driven by the hydrophobic residues Y28α, L30α and M98β. Y28α makes additional van der Waals contacts to CD1c Glu61 and Glu62, L30α to Glu157 and M98β to Tyr152 (**Figures 2B, D, H**). In contrast, G94α loses few contacts to CD1c Asp65 when compared to S2c^WT^. Overall, the significant increase in hydrophobic interactions not only increases the affinity to towards CD1c but also to the lipid selectivity primarily through S30β and M98β.

### Mass spectrometry analysis reveals TCR preference for lipids with larger head group

As structural information showed likely differences in the lipid selectivity between S2c^WT^ and S2c^a5b6^ when bound to CD1c-endo, we investigated the nature of lipids present in these two complexes using mass spectrometry.

**Figure 4:**
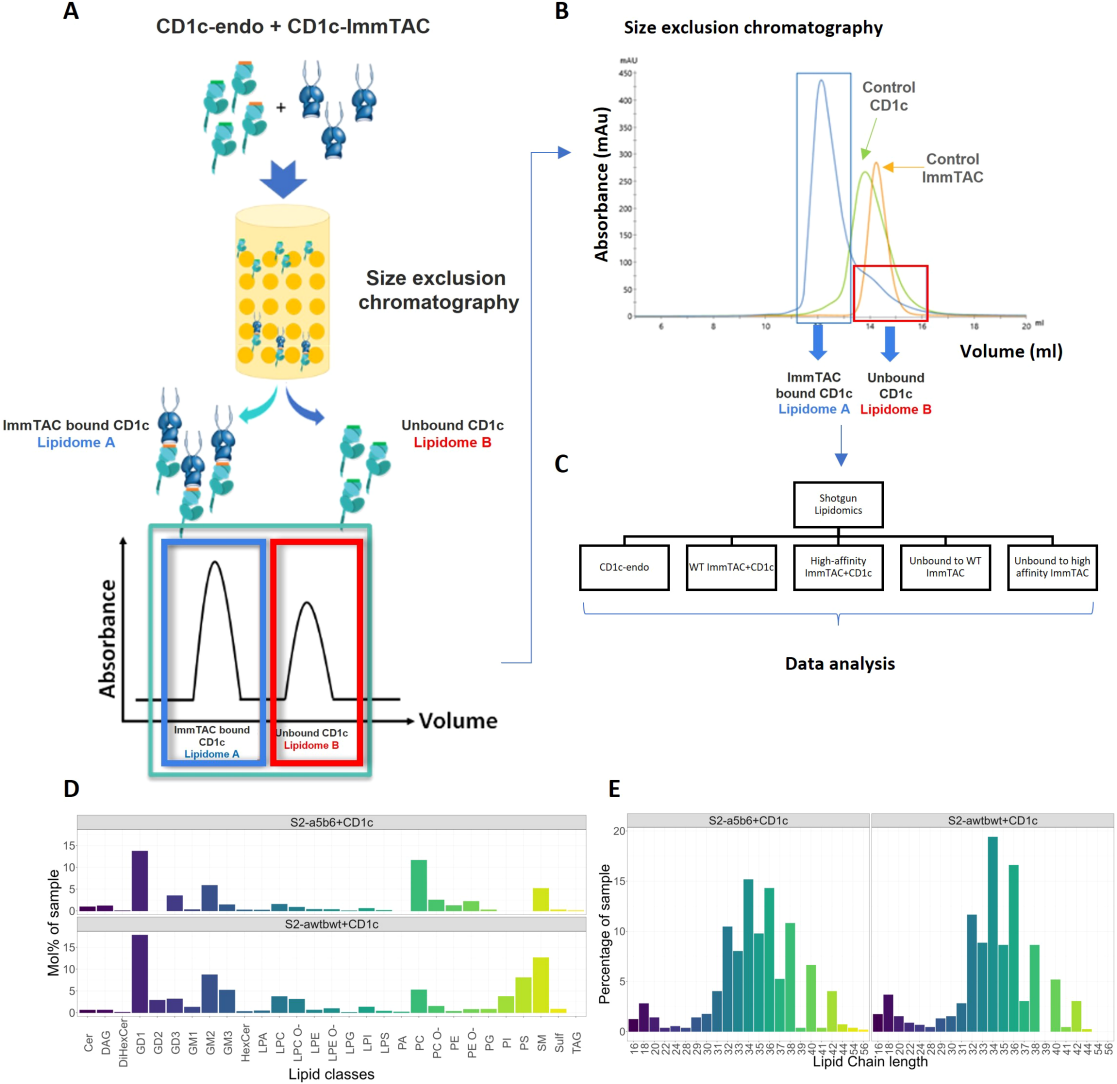
Lipidomic analysis of CD1c-lipid complexes trapped by CD1c–specific ImmTACs. **(A)** CD1c-endo molecules expressed in Expi293F cells were complexed with CD1c-specific ImmTACs, the CD1c-ImmTAC complex and the unbound CD1c fractions were separated by size exclusion chromatography. **(B)** CD1c-ImmTAC mixes were loaded onto size exclusion chromatography column and the separation was monitored by ultraviolet light and gel electrophoresis to determine the CD1c to ImmTAC ratio, and allowing normalization of lipid eluents based on protein abundance. **(C)** Shotgun lipidomics and data analysis were carried out comparing CD1c-endo, WT and High affinity ImmTAC+CD1c complexes and unbound fractions. HLA-A2 and ImmTACs alone were used as background. **(D)** Lipid composition of S2c^WT^-CD1c and S2c^a5b6^-CD1c complexes. **(E)** Lipid chain length profile of the CD1c-associated lipid ligands recognised by the phage-isolated low affinity TCR and affinity enhanced ImmTACs.

Unbound CD1c molecules contain endogenous lipids from the Expi293F cell line and were purified using detergent-free methods to avoid the loss of lipids. Soluble S2c^WT^ and S2c^a5b6^ TCRs were expressed in ExpiCHO cells. CD1c-endo molecules were mixed with each TCR at a 1:1 ratio and complexes were purified by Size Exclusion Chromatography (**Figure 4A**). Lipids from TCR-CD1c-endo complexes and unbound CD1c-endo were extracted for mass spectrometry analysis (**Figures 4B, C**). We detected 402 shared lipid features in the TCR-CD1c co-purification samples, including ceramides, glycosphingolipids, phospholipids, lyso-lipids and glyco-lipids, indicating consistent lipid repertoires between the phage-isolated low-affinity and affinity-enhanced TCR-CD1c complexes (**Figure 4D**). Both complexes retained phospholipids as the most abundant species, with a shift in chain-length profiles toward larger lipids in the affinity-enhanced complex. Similar acyl-chain pairings were observed for PC and PE lipids in both complexes, while PI and PS species with similar acyl-chain lengths were present in the low-affinity complex but were absent from the high-affinity complex. This suggests that the selectivity of the high-affinity TCR is influenced more by the lipid headgroups occupying the A’ pocket than by acyl-chains buried within the F’ pocket. Notably, the lipids absent in the high-affinity complex (e.g., PI and PS) exhibited negatively charged headgroups.

Certain larger lipids, such as TAG 54:1;0, TAG 54:2;0, TAG 56:2;0, and GM2 42:2;2, were exclusively bound to the high-affinity TCR-CD1c complex, highlighting potential differences in the dynamics of the TCR-CD1c interface. Additionally, short-chain lyso-lipids (C16-C28) were identified in both complexes and may accommodate within the F’ pocket of the CD1c binding groove. These short-chain lipids (C16-C18) might act as spacers stabilizing the CD1c groove and aid the presentation of other lipids occupying the F’ pocket.

Altogether, the mass spectrometry data suggest that the affinity maturation of the S2c^WT^ TCR primarily enhanced interactions with CD1c heavy chains rather than significantly altering the repertoire of recognised lipids. Larger lipids and short-chain lyso-lipids unique to the high-affinity complex may reflect subtle alterations in the TCR-CD1c interface, emphasizing the intricate interplay between TCR affinity and lipid presentation.

### T-cell redirection against CD1c positive cells using a TCR-CD3 bispecific

One high affinity CD1c specific TCR variant (S2c^a5b6^) was reformatted as bispecific molecules by fusing it to an anti-CD3 scFv to produce immune mobilising monoclonal TCRs against cancer (ImmTACs, **Supplementary Figure S4C**) (44). To evaluate the potency of ImmTACs against CD1c positive and negative cell lines, β2m and CIITA genes were knocked out from the THP1 wild-type cell line (acute monocytic leukemia) using CRISPR/Cas9 (THP-DKO), abrogating cell surface expression of MHC class I and class II molecules (18). THP1-DKO cells were transduced with single chain CD1c-β2m gene constructs (THP1-CD1c) or single chain CD1b-β2m gene constructs (THP1-CD1b) using lentiviral particles, to obtain constitutive cell surface expression of CD1c or CD1b molecules. As additional specificity controls, the C1R cell line (human B-cell lymphoblastoid line) was transduced with CD1c-β2m or CD1d-β2m and the K562 (chronic myelogenous leukemia) cell line was transduced with CD1c-β2m. Cells were stained with anti-CD1c antibody (L161 clone) to confirm CD1c expression levels by FACS, which was high on THP1-CD1c, K562-CD1c, and C1R-CD1c, undetectable on THP1, C1R and K562 parental cells and negative as expected on THP1-DKO (**Supplementary Figure S4A**).

**Figure 5:**
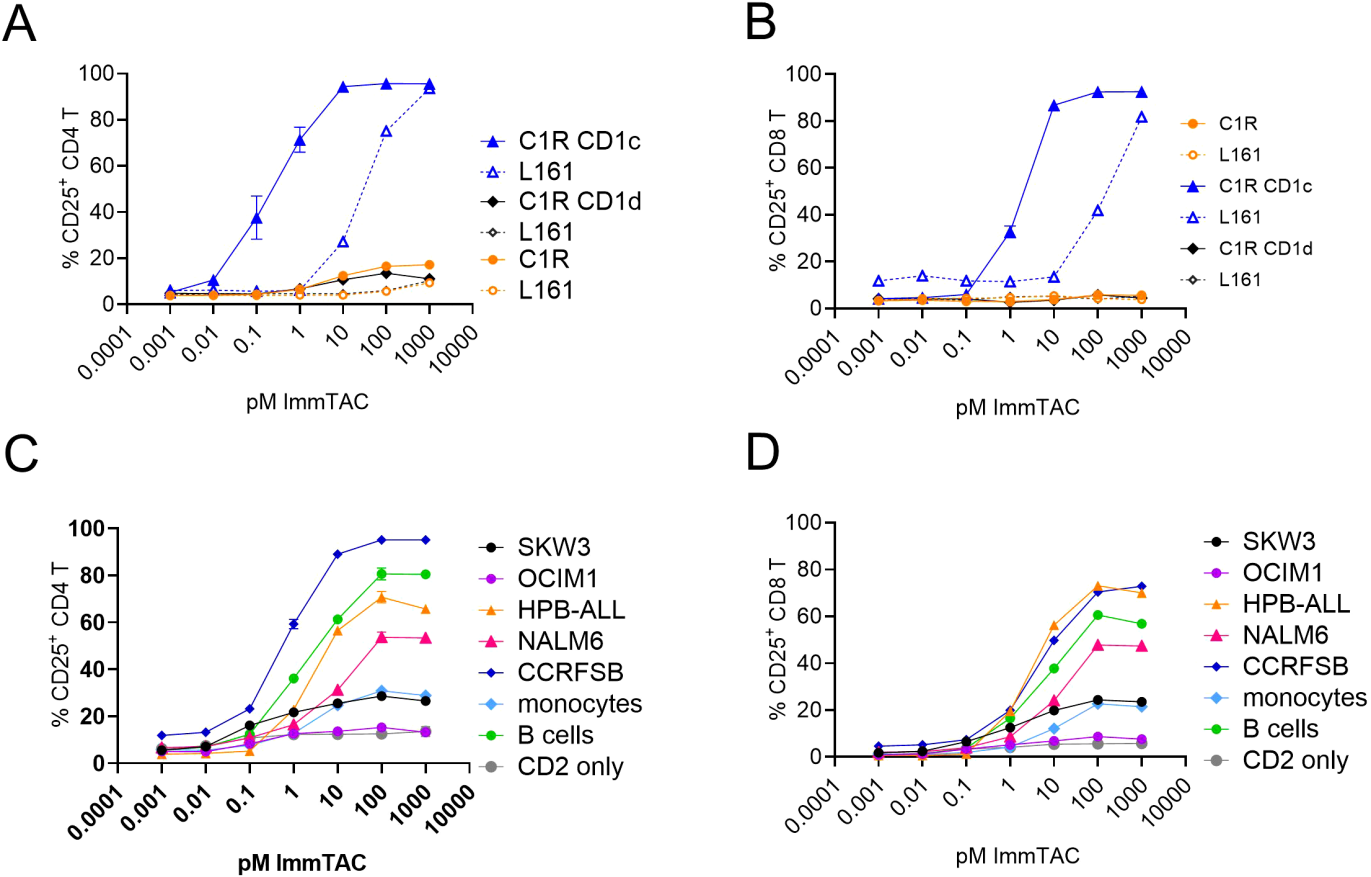
CD1c restricted ImmTAC molecules activate pan T-cells against cancer cell lines expressing variable levels of CD1c. CD2 enriched T-cells were co-cultured with the indicated cancer cell lines to assess potency of the S2c-^a5b6^ ImmTAC molecule. **(A, B)** Dose response curve of CD4 **(A)** or CD8 **(B)** T cell activation to C1R, C1R CD1c or C1R CD1d targets. **(C, D)** Dose response curve of CD4 **(C)** or CD8 **(D)** T cell activation to SKW3, OCIM1, HPB-ALL, NALM6, CCRFSB, monocytes and B cells. CD2 cells in the absence of targets are also depicted. Panels A and B depict the percentage of CD25 expressing T cells after overnight activation in pre presence (dotted lines) or absence (solid lines) of anti-CD1d blocking antibody L161. The blocking data relative to panels **(C, D)** are shown in **Supplementary Figure S5**. One experiment of two, performed in triplicates.

The ImmTAC potently activated CD4 and CD8 T-cells against CD1c positive cell lines as assessed by CD25 upregulation (**Figures 5A, B**, **Supplementary Figures S5A–D**). Additionally, T cell activation was partially blocked by the L161 anti-CD1c antibody (**Figures 5A, B**, **Supplementary Figures S5A–D**). We next assessed the ability of the S2c^a5b6^ ImmTAC to redirect T cells against a panel of haematological cell lines (**Supplementary Table S1**) with various levels of CD1c expression (**Supplementary Figure S4B**). We observed T cell activation against CCRFSB, NALM6, HPB-ALL, SKW3 but not OCIM1 (**Figures 5C, D**), and specificity was confirmed by almost complete blockade in the presence of the L161 anti-CD1c antibody (**Supplementary Figures S5G, H**). Consistently with the lipid agnostic mode of recognition of the S2c^a5b6^ ImmTAC, we observed reactivity to primary B cells and – to a lesser extent - monocytes, which could be specifically blocked by the L161 anti-CD1c antibody (**Figures 5C, D**, **Supplementary Figures S5E, F**).

These results suggest that ImmTAC molecules targeting CD1c molecules could be developed for immunotherapy of haematologic tumours. Further engineering however would be required to obviate the on-target off-tumour effect against primary monocytes and B cells.

## Discussion

The non polymorphic, MHC-like CD1 molecules present self and microbial lipids to subsets of T cells bearing αβ and γδ TCRs. Since the original identification of CD1-restricted T cells in 1989 (45), we have a deeper understanding of the repertoire of lipids presented by different isoforms (29, 46), the molecular mechanisms of lipid antigen presentations (47) and the breadth of T cell responses (48). The monomorphic nature of CD1 molecules, the limited tissue expression and the conserved nature of lipids, make the CD1 axis attractive to develop universal cancer immunotherapies (49).

In this study, we demonstrate the feasibility of targeting CD1c in leukemic cells through a lipid agnostic bispecific T cell engager, with an affinity enhanced soluble TCR as the targeting arm. We isolated a CD1c-restricted TCR (S2c) from phage TCR libraries and showed that it bound to CD1c-endo with broad lipid selectivity, a finding supported by the crystal structure. We used a combination of structurally informed selected interface residues and pan-CDR mutational library approaches to generate an affinity enhanced variant of S2c, S2c^a5b6^. Crystal structure of S2c^a5b6^-CD1c-endo complex revealed the molecular mechanism underpinning enhanced affinity and lipid selectivity, especially at the F’-portal region. While several CD1b and CD1d trimolecular structures have been solved by x-ray crystallography (38), we report the second structure of an autoreactive CD1c-restricted TCR complex. We observed some similarities in the CD1c interaction footprint of the S2c^WT^ and S2c^a5b6^ TCRs to the previously reported CD1c-restricted 3C8 TCR (31). Unlike CD1c-restricted TCRs that exhibit high specificity for mycobacterial phosphomycoketide antigens, imparted through unique CDR loop residues (8), the self-reactive CD1c-restricted 3C8 TCR achieved polyspecificity through binding centrally on the A’ roof of CD1c, with the TCRβ chain blocking the protrusion of lipid(s) through the F’ portal (31). In our structure, and possibly because of a slightly different docking angle, while most of the S2c^WT^ TCR contacts were focused over the CD1c A’-roof, the F’ portal was not blocked. Flexibility of the residues Q95α and Y48β was observed between the two asymmetric units, paralleled by two different side chain conformations adopted by CD1c F67, to accommodate larger lipid head groups. In the high affinity S2c^a5b6^ TCR, mutations in the TCR residues S30β and M98β created additional space to accommodate larger lipid head groups and wrap around the lipid density, significantly differing from the 3C8 TCR. This binding mode is more reminiscent of CD1b-TCR published structures (50) and of the open CD1c F’ conformations seen in the CD1c-(mannosyl)phosphomycoketide structures (7, 8). These findings suggest that while small lipid size plays a crucial role in determining autoreactive T cell responses (48), tuning the TCR binding affinity through mutagenesis refines the interplay between the TCR and lipids bound to the CD1c groove. Accordingly, mass spectrometry analysis of lipids purified from the S2c^a5b6^-CD1c complex via a TCR trap system (51) revealed enrichment of larger lipids, such as TAG 54:1;0, TAG 54:2;0, TAG 56:2;0, and GM2 42:2;2. Additionally, by affinity enhancing the TCR, we achieved binding to 100% of the CD1c-endo molecules, while the S2c^WT^ TCR (K_D_= 16.7 μM) and the 3C8 TCR (K_D_= 40 μM) bound to 75% and 15-20%, respectively (31).

CD1-specific TCR recognition studies suggest that most T cells recognise CD1 lipid complexes via co-recognition of both ligand and antigen presenting molecule, similarly to the MHC-peptide recognition concept (52, 53). Indeed, invariant natural killer T cell receptors (iNKT TCRs) contact both CD1d and the protruding lipid-headgroups (54), similarly to CD1b-specific TCRs, that make contact with the CD1b heavy chain and the accessible lipid headgroups (34, 50). Conversely, the crystal structure of a CD1a autoreactive TCR revealed direct recognition of CD1a over the A’ roof, in a lipid agnostic manner (35). The crystal structure of the self-reactive 3C8 CD1c-restricted TCR revealed a more central footprint, sequestering the lipid within the CD1c cleft (31). The trimolecular complex of our S2c TCR reveals yet another modality of docking of an autoreactive CD1c TCR, which retains polyspecificity but can also recognise larger lipid head groups. Altogether, except for the autoreactive γδ TCR with an atypical sideways recognition of CD1a molecule (55), most CD1-restricted TCR binding modes published to date are broadly analogous to classical TCR-pHLA complexes (53). The TCR binds on top of the CD1 binding groove, with the complementarity determining region (CDR) loops positioned with the potential to make contacts with the surface of CD1 and the exposed lipid head group. Apart from selected CD1d restricted αβ TCRs which utilise an ‘unconventional’ parallel crossing angle with respect to the CD1 binding groove during engagement (53), all other CD1 restricted TCRs utilise a ‘canonical’ diagonal crossing angle to engage their respective antigens (31, 34–36) (**Supplementary Figure S2**).

CD1c reactive T cells have been detected at high frequency in human blood (56, 57) expand during tuberculosis infection (58, 59) and might have a pathogenic role in autoimmune disease (60), and CD1c expression is modulated by inflammatory cytokines (61). While the full TCR repertoire of CD1c-reactive T cells remain to be determined, it encompasses αβ and γδ TCRs (36, 37, 56, 62). To date, there is no evidence for restricted TCR chain usage by CD1c-restricted T cells, unlike what is observed for the semi-invariant CD1d-restricted iNKT TCR (63) and biased TRBV4-1 representation in CD1b-restricted T cells (64). Yet, several autoreactive CD1c-restricted TCRs also bear the TRBV4.1 gene segment, albeit with different junctional segments and CDR3 lengths (56, 62). Mycobacterial-reactive CD1c-restricted TCRs isolated from different donors expressed TRBV7.9 (8). Interesting, the S2c TCR we identified from phage libraries also used the TRBV7.9 gene segment, differing in that from the TRBV7.2 3C8 TCR. However, we have not assessed whether the S2c TCR is cross-reactive to phosphomycoketide, thus explaining the ability to bind open conformations of CD1c-lipid complexes. This would not be unexpected, as it has been shown that self-reactive CD1a, CD1b and CD1c restricted T cells can have dual reactivity to foreign microbial antigens (65).

Bispecific engagers and bispecific antibodies have revolutionised the treatment of hematological malignancies of B cell lineage (multiple myeloma, non-Hodgkin lymphoma and ALL), with 7 FDA approved products (66). While off-tumor on-target toxicities are observed, they can be managed. Limited progress has been made for myeloid malignancies, because targeting shared surface antigens between leukemic cells and hematopoietic stem cells can lead to myeloablation. CD1c molecules are expressed on blast cells of 54% of adult patients with AML and 45% of paediatric patients, but not on normal hematopoietic cells therefore are a promising monomorphic therapeutic target (11). We engineered the high affinity TCR in a bispecific engager format, with an anti CD3 effector arm. We demonstrated on-target specificity while achieving high potency. However, the lipid agnostic feature of this TCR confers reactivity to CD1c positive primary cells, such as B cells and monocytes, limiting its therapeutic applications. Further understanding of the lipidome of malignant leukemic cells and identification of tumour specific lipid antigens will allow to develop lipid-specific TCR-based T cell engagers a new class of off-the shelf immunotherapeutic for CD1c positive leukemias.

## Data Availability

The original contributions presented in the study are included in the article/**Supplementary Files**, further inquiries can be directed to the corresponding author/s.

## Supplementary material

Supplementary Figure 1 S2c^WT^ TCR binding affinity and affinity-enhancement. Binding dissociation constant (affinity) of the **(A)** S2c^WT^ TCR and **(B)** affinity-enhanced variants interacting with CD1c loaded with endogenous lipids from HEK cells was measured using surface plasmon resonance. The estimated % of CD1c-endo bound by the TCR was calculated by dividing the observed Rmax by the theoretical Rmax based on the amount of CD1c loaded. Data representative of three experimental repeats with different preparations of CD1c-endo.

Supplementary Figure 2 Structural overview of CD1-TCR complexes. **(A)**. Overlay of the two CD1a-endo-TCR complexes. The gamma-delta TCR (green) was shown to bind in a lipid independent orientation. **(B)**. Overlay of CD1b-TCR complexes in presence of specific lipids. CD1b binding TCRs broadly adopt similar docking orientation. **(C)**. Overlay of selected human CD1d-TCR complexes. Binding orientation of CD1d restricted TCRs broadly falls into two different categories.

Supplementary Figure 3 Affinity-enhanced S2c TCR cross allele reactivity screening. Affinity-enhanced S2c TCRs were screened against CD1a, CD1b, CD1d, and various different peptides in complex with HLA-A2, HLA-A11, HLA-B7, HLA-B35 and HLA-Cw304 using surface plasmon resonance. 10 nM of each ImmTAC was injected and binding was measured against each HLA monomers. **(A)** Binding curves of S2c TCRs against CD1 isoforms and HLA-peptide mixes. **(B)** CD1 isoforms and HLA-peptide mixes used for cross-reactivity screening.

Supplementary Figure 4 CD1 expression levels on cancer cell lines used in ImmTAC assays **(A)** Histograms depicting expression of CD1b, CD1c or CD1d on the indicated cell lines. Gray, isotype control; orange CD1 staining. **(B)** Histograms depicting expression of CD1c on the indicated cell lines. Gray, isotype control; orange CD1 staining. **(C)** Cartoon depicting an ImmTAC molecule. Soluble extracellular domains of TCRα and β chains are stabilized by an additional disulphide bond and the N-term of the TCRβ chain is fused to the anti-CD3scFv via a single GS linker (see Methods).

Supplementary Figure 5 The specificity and cross-reactivity of the S2c^a5b6^ ImmTACs molecules to activate pan T-cells. CD2 enriched T-cells were co-cultured with the indicated cancer cell lines to assess potency of the S2c^a5b6^ ImmTAC molecule. **(A, B)** Dose response curve of CD4 **(A)** or CD8 **(B)** T cell activation to K562 or K562 CD1c targets. **(C, D)** Dose response curve of CD4 **(C)** or CD8 **(D)** T cell activation to THP1, THP1 DKO, THP1 CD1b and THP1 CD1c targets. **(E, F)** Dose response curve of CD4 **(E)** or CD8 **(F)** T cell activation to monocytes and B cells. CD2 cells in the absence of targets are also depicted. **(G, H)** Dose response curve of CD4 **(G)** or CD8 **(H)** T cell activation to SKW3, OCIM1, HPB-ALL, NALM6, CCRFSB and MOLT4. All panels depict the percentage of CD25 expressing T cells after overnight activation in pre presence (dotted lines) or absence (solid lines) of anti-CD1c blocking antibody L161. This figure complements **Figure 5**. One experiment of two, performed in triplicates.
