# Supplementary figures and images for: A CD1c lipid agnostic T cell receptor bispecific engager redirects T cells against CD1c+ cells
Source: Front Immunol. 2025 Jul 24;16:1614610. doi: 10.3389/fimmu.2025.1614610 (PMC12328196; doi:10.3389/fimmu.2025.1614610)

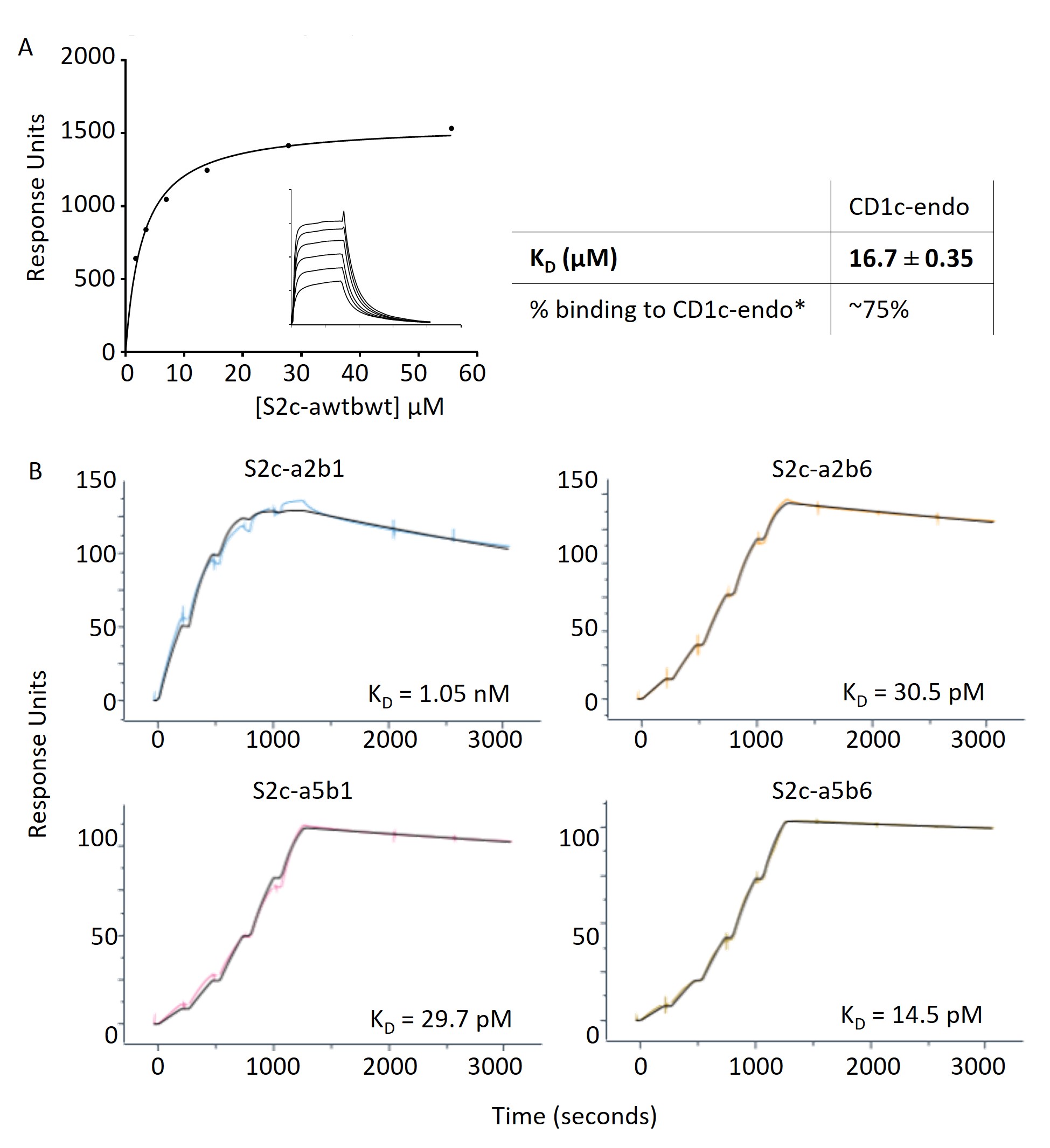

Supplement: Supplementary Figure 1 — S2cWT TCR binding affinity and affinity-enhancement. Binding dissociation constant (affinity) of the (A) S2cWT TCR and (B) affinity-enhanced variants interacting with CD1c loaded with endogenous lipids from HEK cells was measured using surface plasmon resonance. The estimated % of CD1c-endo bound by the TCR was calculated by dividing the observed Rmax by the theoretical Rmax based on the amount of CD1c loaded. Data representative of three experimental repeats with different preparations of CD1c-endo. [file Image1.jpeg]

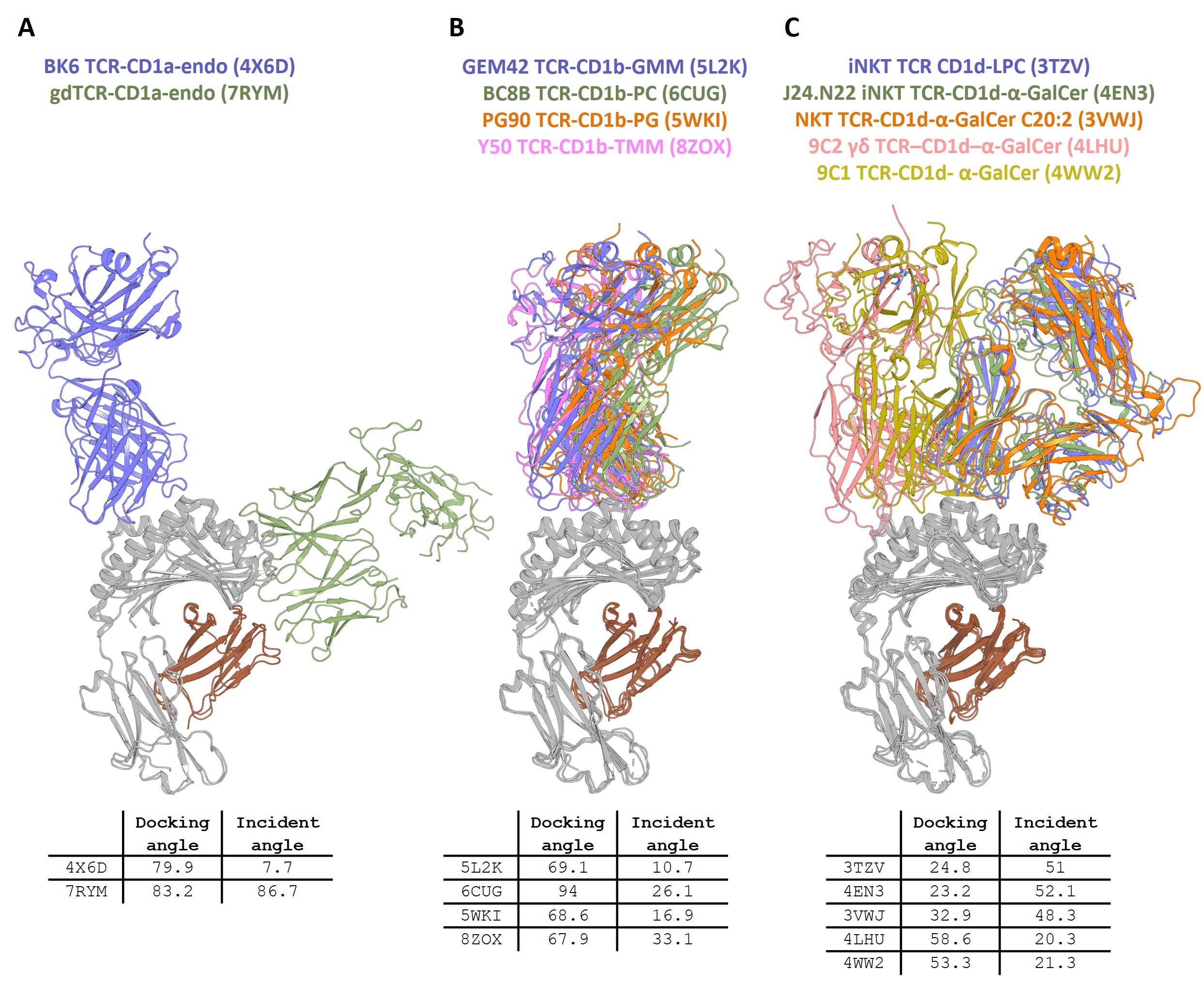

Supplement: Supplementary Figure 2 — Structural overview of CD1-TCR complexes. (A). Overlay of the two CD1a-endo-TCR complexes. The gamma-delta TCR (green) was shown to bind in a lipid independent orientation. (B). Overlay of CD1b-TCR complexes in presence of specific lipids. CD1b binding TCRs broadly adopt similar docking orientation. (C). Overlay of selected human CD1d-TCR complexes. Binding orientation of CD1d restricted TCRs broadly falls into two different categories. [file Image2.jpeg]

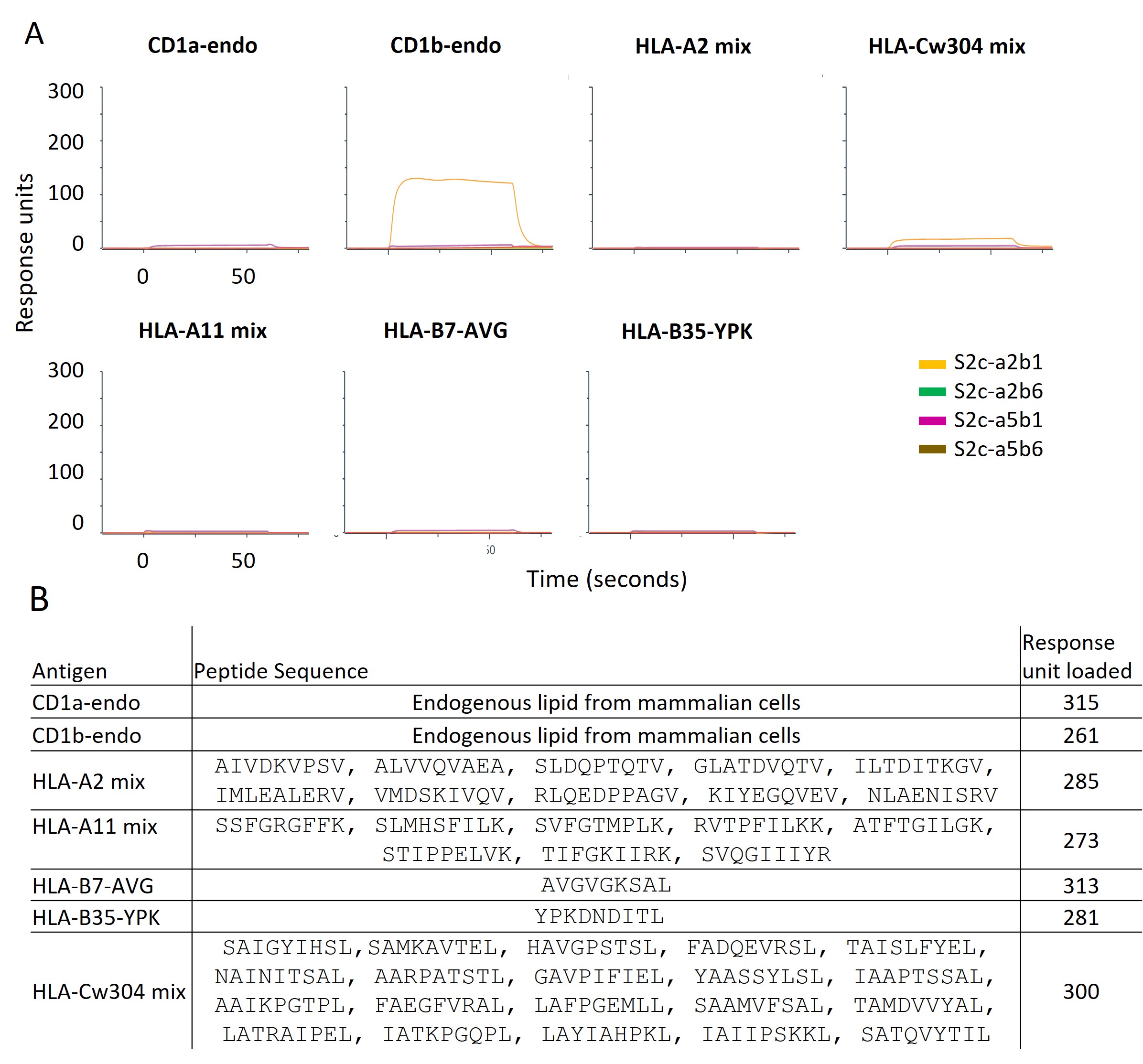

Supplement: Supplementary Figure 3 — Affinity-enhanced S2c TCR cross allele reactivity screening. Affinity-enhanced S2c TCRs were screened against CD1a, CD1b, CD1d, and various different peptides in complex with HLA-A2, HLA-A11, HLA-B7, HLA-B35 and HLA-Cw304 using surface plasmon resonance. 10 nM of each ImmTAC was injected and binding was measured against each HLA monomers. (A) Binding curves of S2c TCRs against CD1 isoforms and HLA-peptide mixes. (B) CD1 isoforms and HLA-peptide mixes used for cross-reactivity screening. [file Image3.jpeg]

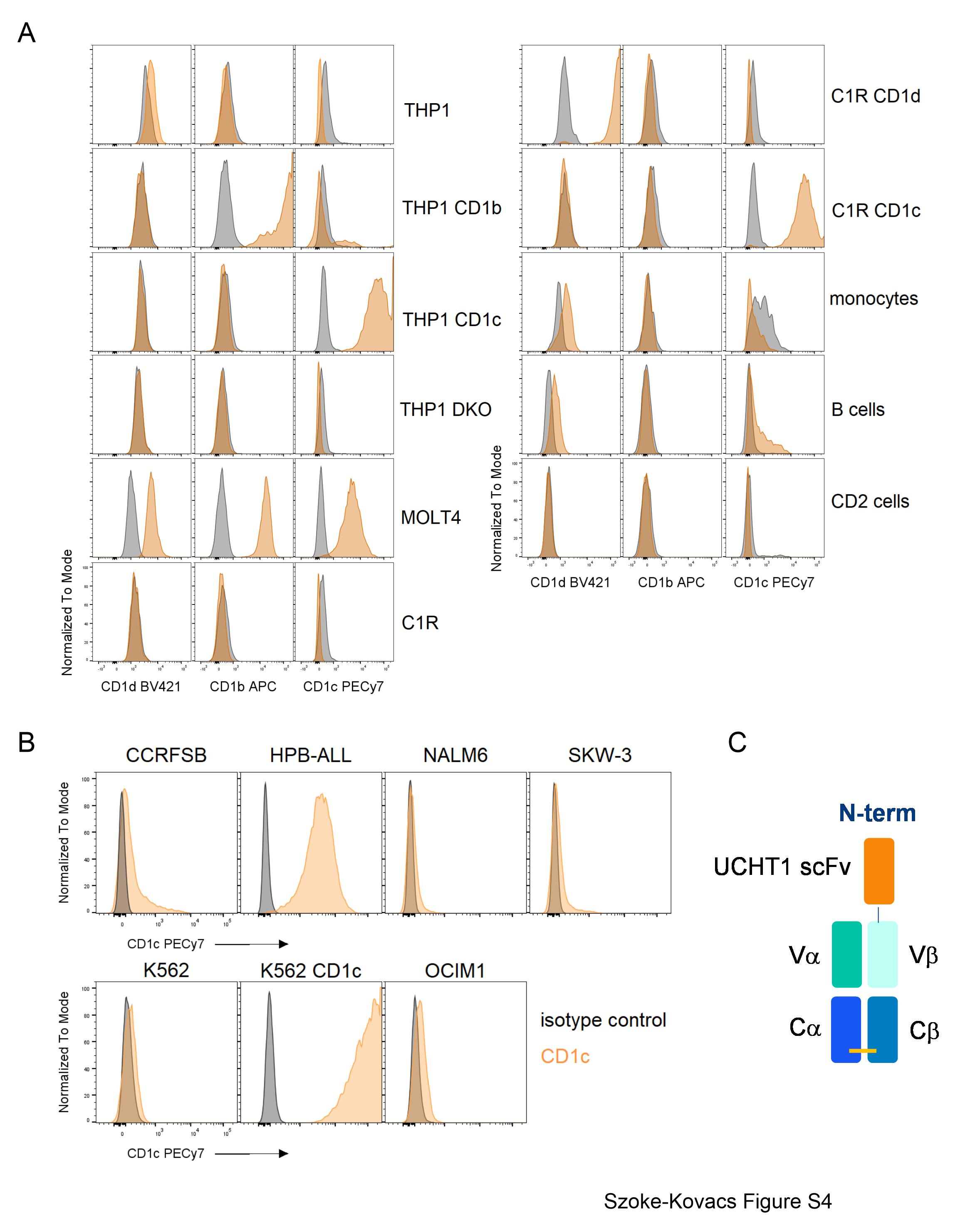

Supplement: Supplementary Figure 4 — CD1 expression levels on cancer cell lines used in ImmTAC assays (A) Histograms depicting expression of CD1b, CD1c or CD1d on the indicated cell lines. Gray, isotype control; orange CD1 staining. (B) Histograms depicting expression of CD1c on the indicated cell lines. Gray, isotype control; orange CD1 staining. (C) Cartoon depicting an ImmTAC molecule. Soluble extracellular domains of TCRα and β chains are stabilized by an additional disulphide bond and the N-term of the TCRβ chain is fused to the anti-CD3scFv via a single GS linker (see Methods). [file Image4.jpeg]

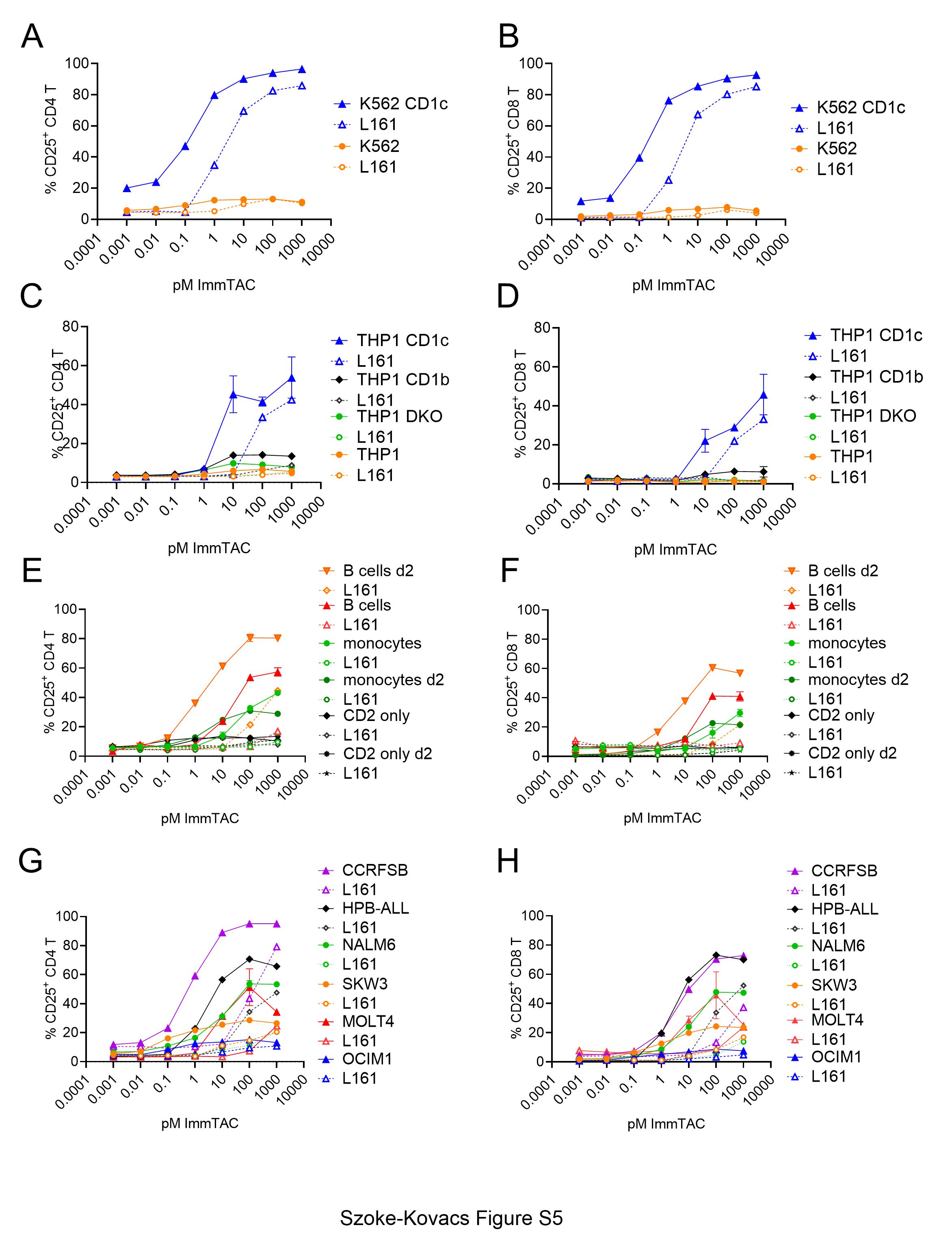

Supplement: Supplementary Figure 5 — The specificity and cross-reactivity of the S2ca5b6 ImmTACs molecules to activate pan T-cells. CD2 enriched T-cells were co-cultured with the indicated cancer cell lines to assess potency of the S2ca5b6 ImmTAC molecule. (A, B) Dose response curve of CD4 (A) or CD8 (B) T cell activation to K562 or K562 CD1c targets. (C, D) Dose response curve of CD4 (C) or CD8 (D) T cell activation to THP1, THP1 DKO, THP1 CD1b and THP1 CD1c targets. (E, F) Dose response curve of CD4 (E) or CD8 (F) T cell activation to monocytes and B cells. CD2 cells in the absence of targets are also depicted. (G, H) Dose response curve of CD4 (G) or CD8 (H) T cell activation to SKW3, OCIM1, HPB-ALL, NALM6, CCRFSB and MOLT4. All panels depict the percentage of CD25 expressing T cells after overnight activation in pre presence (dotted lines) or absence (solid lines) of anti-CD1c blocking antibody L161. This figure complements Figure 5 . One experiment of two, performed in triplicates. [file Image5.jpeg]
