# Supplementary material for: A CD1c lipid agnostic T cell receptor bispecific engager redirects T cells against CD1c+ cells
Source: Front Immunol. 2025 Jul 24;16:1614610. doi: 10.3389/fimmu.2025.1614610 (PMC12328196; doi:10.3389/fimmu.2025.1614610)
Supplement: Supplementary file 6 [file Table1.docx]

| **Cell line** | **Indication** | **Vendor** | **Cat. number** |
| --- | --- | --- | --- |
| THP1 | Acute monocytic leukemia (AML) | ATCC | TIB-202 ™ |
| C1R | Lymphoblastoid cell line | (Suckling et al., 2025) Frontiers in Immunology, 16, 1547664. |  |
| K562 | Chronic myeloid leukemia (CML) | DSMZ/ATCC | DSMZ (ACC 10)/ ATCC (CCL 243) |
| MOLT4 | Acute lymphocytic leukemia (ALL) | ECACC | 85011413 |
| CCRFSB | Acute lymphocytic leukemia (ALL) | ATCC | CCL 120 |
| HPB-ALL | Acute lymphocytic leukemia (ALL) | DSMZ | ACC 438 |
| NALM6 | Acute lymphocytic leukemia (ALL) | DSMZ | ACC 128 |
| OCIM1 | Acute monocytic leukemia (AML) | DSMZ | ACC529 |
| SKW-3 | Chronic lymphocytic leukaemia (CLL) | DSMZ | ACC 53 |

**Table S1:** **Cancer** **Cell lines**

Cancer cell lines used in experiments.
