# Supplementary material for: A CD1c lipid agnostic T cell receptor bispecific engager redirects T cells against CD1c+ cells
Source: Front Immunol. 2025 Jul 24;16:1614610. doi: 10.3389/fimmu.2025.1614610 (PMC12328196; doi:10.3389/fimmu.2025.1614610)
Supplement: Supplementary file 7 [file Table2.docx]

| ***PDB CODE*** | **9QWJ** | **9QWK** |
| --- | --- | --- |
| ***Molecule*** | S2c^WT^-CD1c complex | S2c^a5b6^-CD1c complex |
| *Space group* | P 1 21 1 | P1 |
| *Unit cell dimensions* | a=85.56, b=121.93, c=109.07; α=90, β=110.09, γ=90 | a=82.23, b=83.65, c=180.13; α=91.85, β=95.99, γ=115.61 |
| *X-ray source* | DLS I03 | DLS I04 |
| *Wavelength (Å)* | 0.9762 | 0.9537 |
| *Resolution range (Å)* | 102.43-2.04 (2.30-2.04) | 89.24 - 2.27 (2.31 - 2.27) |
| *Observations* | 693142 (32351) | 706617 (35372) |
| *Unique reflections* | 87692 (4386) | 194208 (9525) |
| *Multiplicity* | 7.9 (7.4) | 3.6 (3.7) |
| *Completeness (%)* | 93.2 (61.9) | 98.52 (96.77) |
| *Mean I/σ( I)* | 10.6 (1.7) | 14.6 (0.8) |
| *R_merge_* | 0.135 (1.205) | 0.101 (1.660) |
| *R_meas_* | 0.145 (1.294) | 0.119 (1.943) |
| *R_pim_* | 0.051 (0.468) | 0.063 (1.004) |
| *CC_1/2_* | 0.998 (0.668) | 0.963 (0.325) |
| ***Refinement*** |  |  |
| *Rwork / Rfree (%)* | 20/23.5 | 22.6/25.3 |
| *RMS (bonds)* | 0.0091 | 0.0079 |
| *RMS (angles)* | 1.779 | 1.630 |
| *Mean B value (Å^2^)* | 42.43 | 72.91 |
|  |  |  |

^a^Values in the parentheses refer to the outer resolution shell

**Table S2: X-ray data collection and refinement statistics**

^aValues in the parentheses refer to the outer resolution shell^

**Table S1: X-ray data collection and refinement statistics**
