# Supplementary material for: A CD1c lipid agnostic T cell receptor bispecific engager redirects T cells against CD1c+ cells
Source: Front Immunol. 2025 Jul 24;16:1614610. doi: 10.3389/fimmu.2025.1614610 (PMC12328196; doi:10.3389/fimmu.2025.1614610)
Supplement: Supplementary file 8 [file Table3.docx]

| **CD1c** | **S2c^WT^** | | **S2c^a5b6^** | |
| --- | --- | --- | --- | --- |
| Ser59 | Thr28α | 1 |  |  |
|  |  |  | Tyr28α | 1HB |
| Glu61 |  |  | Tyr28α | 4 |
| Glu62 | Thr28α | 5, 1HB | Tyr28α | 10 |
| Asp65 | Asp94α | 5 | Gly94α | 2 |
|  | Gln95α | 9, 2HB | Gln95α | 8, 4HB |
|  | Tyr96α | 2 | Tyr96α | 2 |
| Leu66 | Tyr96α | 1 | Tyr96α | 3 |
| Leu68 | Gln95α | 4 | Gln95α | 5 |
|  | Glu56β | 1 | Glu56β | 3 |
| Leu69 | Gln95α | 3 | Gln95α | 2 |
|  | Tyr96α | 2 | Tyr96α | 1 |
|  | Trp98α | 1 | Trp98α | 1 |
| Arg71 | Leu55α | 3, 2HB | Leu55α | 2, 2HB |
| Phe72 | Gln95α | 1 | Gln95α | 2 |
|  | Tyr48β | 5 | Tyr48β | 6 |
|  | Gln50β | 7, 1HB | Gln50β | 5, 1HB |
|  | Leu55β | 1 | Leu55β | 3 |
|  | Glu56β | 1 | Glu56β | 1 |
| Phe75 | Gln50β | 3 | Gln50β | 2 |
|  | Asn51β | 1 | Asn51β | 1 |
|  | Glu52β | 1 | Glu52β | 1 |
|  | Ala53β | 2 | Ala53β | 2 |
|  | Leu55β | 2 | Leu55β | 1 |
| Gly76 | Gln50β | 1, 1HB | Gln50β | 2 |
| Arg79 | Asn51β | 3 | Asn51β | 2 |
|  | Glu52β | 4, 3HB, 1SB | Glu52β | 4, 3HB, 1SB |
| Glu80 | Arg97β | 3, 3HB, 1SB | Arg97β | 5, 3HB, 1SB |
| Tyr152 | Arg97β | 1HB | Arg97β | 1HB |
|  |  |  | Met98β | 1 |
| Gly154 | Trp98α | 2 | Trp98α | 2 |
|  |  |  | Arg100β | 2 |
| Val155 |  |  | Trp98α | 1 |
| Glu157 |  |  | Leu30α | 3 |
|  | Lys52α | 3, 1HB, 1SB | Lys52α | 1, 1HB, 1SB |
|  | Arg100β | 1, 1HB | Arg100β | 3 |
| Thr158 | Tyr96α | 2 | Tyr96α | 1 |
|  | Trp98α | 3, 1HB | Trp98α | 4, 1HB |
|  | Arg100β | 2 | Arg100β | 1 |
| Asn161 | Tyr30α | 3 | Leu30α | 4 |
|  |  |  | Tyr96α | 2 |
| Leu162 | Tyr96α | 4 | Tyr96α | 4 |
| Ser165 | Tyr30α | 1, 1HB |  |  |
| Thr166 | Tyr30α | 1 |  |  |
|  | Tyr96α | 3, 1HB | Tyr96α | 4, 1HB |

**Supplementary table S3**: List of contacts between S2cWT/S2c^a5b6^ TCR and CD1c. Van der Waals, hydrogen bond (HB) and salt bridge (SB) contacts up to 4Å distance are listed. Interface mutated residues in S2c^a5b6^ are highlighted in red.
