# Supplementary material for: A CD1c lipid agnostic T cell receptor bispecific engager redirects T cells against CD1c+ cells
Source: Front Immunol. 2025 Jul 24;16:1614610. doi: 10.3389/fimmu.2025.1614610 (PMC12328196; doi:10.3389/fimmu.2025.1614610)
Supplement: Supplementary file 9 [file Table4.docx]

| **CD1c** | **3C8 TCR (PDB 6C09)** | |
| --- | --- | --- |
| Glu62 | Ser28α | 3, 1HB |
|  | Met29α | 3 |
| Asp65 | Asn27α | 3, 1HB |
|  | Val108α | 1 |
|  | Lys111α | 2, 1HB, 1SB |
| Leu66 | Met29α | 2 |
| Leu68 | Val108α | 1 |
|  | Asp110α | 2 |
|  | Lys111α | 3 |
| Leu69 | Gly109α | 2 |
| Phe72 | Gly109α | 1 |
|  | Asp110α | 5 |
|  | Gln57β | 2 |
|  | Tyr109β | 1 |
|  | Met114β | 1 |
| Phe75 | Gln57β | 16 |
|  | Gly58β | 1 |
| Gly76 | Tyr109β | 2, 1HB |
| Arg79 | Thr30β | 4 |
|  | Tyr109β | 2 |
| Glu80 | Tyr109β | 5, 1HB |
|  | Arg110β | 2, 2HB, 1SB |
| Gln151 | Arg110β | 3, 1HB |
|  | Gly111β | 4, 1HB |
| Tyr152 | Tyr109β | 2 |
|  | Arg110β | 4, 1HB |
| Glu153 | Gly111β | 1HB |
|  | Pro112β | 3 |
| Gly154 | Gly111β | 1, 1HB |
|  | Pro112β | 2, 1HB |
|  | Met114β | 3 |
| Val155 | Tyr109α | 1 |
| Glu157 | Tyr32α | 4, 1HB |
|  | Ser57α | 2, 1HB |
|  | Ile59α | 5 |
|  | Pro112β | 1 |
| Thr158 | Asp31α | 1 |
|  | Met114β | 2 |
| Tyr160 | Ile59α | 3 |
| Asn161 | Asp31α | 1 |
|  | Ser58α | 1, 2HB |
| Thr166 | Met29α | 1 |
| Ser169 | Ser28α | 1, 3HB |
|  | Met29α | 1 |

**Supplementary table 4**: List of contacts between 3C8 TCR and CD1c. Van der Waals, hydrogen bond (HB) and salt bridge (SB) contacts up to 4Å distance are listed.
